# Clinical Presentation, Imaging Features, and Management of Müller–Weiss Disease

**DOI:** 10.7759/cureus.18659

**Published:** 2021-10-11

**Authors:** Shady Hermena, Monica Francis

**Affiliations:** 1 Trauma and Orthopedics, Yeovil District Hospital NHS Foundation Trust, Yeovil, GBR; 2 Department of General Medicine, Dorset County Hospital NHS Foundation Trust, Dorchester, GBR

**Keywords:** müller–weiss disease, paradoxical pes planus varus deformity, navicular bone deformity, midfoot pain, navicular bone fragmentation

## Abstract

Müller-Weiss disease (MWD) is a rare condition of unclear pathogenesis that causes navicular bone collapse and fragmentation. MWD can be challenging to diagnose and presents with midfoot and hindfoot pain and deformities. Although its incidence is unknown, MWD more commonly affects women aged between 40 and 60 years. This study reviews and summarizes the published literature on MWD to allow a better understanding of the pathomechanics, presentation, imaging modalities, and treatment options for MWD.

## Introduction and background

Müller-Weiss disease (MWD) is the idiopathic collapse and fragmentation of the navicular bone that usually manifests with midfoot pain and deformities in adults. MWD should be distinguished from Kohler disease, which is pediatric navicular osteochondrosis [1].

MWD was named after Walther Müller and Konrad Weiss [2-4]. In 1927, Müller originally described this condition as being caused by over-compression of the navicular bone [2]. In the same year, the Austrian radiologist Weiss reported similar findings in two patients [4]. Various hypotheses have been made in the literature to explain MWD pathogenesis; however, its exact pathogenesis remains unclear. This study aimed to review and summarize published evidence of the pathomechanics, clinical presentation, imaging, and treatment of MWD.

## Review

Epidemiology

The actual prevalence and incidence of MWD are difficult to ascertain [5]. MWD commonly affects women aged between 40 and 60 years. Bilateral incidence of MWD is a common finding among the affected population [6]. Maceira and Rochera suggested that MWD has a higher incidence in people who encounter stressful economic and physical circumstances [7]. However, Doyle et al. did not find any specific environmental or social factors to be associated with MWD [8].

Anatomy

The navicular bone has a pyriform shape with proximal concavity and dorsal convexity. Its oblique axis is oriented in the dorsoplantar and lateromedial directions [9]. The navicular bone articulates with the head of the talus proximally, the cuboid inferolaterally, and three cuneiforms distally [9]. It contributes to the medial longitudinal and transverse arches of the foot, and the tibialis tendon inserts into its tuberosity medially [9]. The navicular bone attaches to the two components of the spring ligament (the superomedial and inferior calcaneonavicular ligaments), the dorsal cuneonavicular ligament, the plantar cuneonavicular ligament, the medial cuneonavicular ligament, and the bifurcate ligament (Chopart’s ligament) [9]. The navicular blood supply derives from the medial plantar and dorsalis pedis arteries: the plantar portion is supplied by the medial plantar artery, whereas the dorsal and lateral portions are supplied by the dorsalis pedis artery. Arterial anastomosis between the medial plantar artery and the dorsalis pedis artery supplies the medial navicular tuberosity [9]. The central zone of the navicular bone receives the lowest vascular supply despite being under the maximum shearing stress [10].

Pathomechanics

Despite various theories reported in the literature, the exact etiology of MWD remains unclear. Müller hypothesized that congenital malformations could be the cause of MWD [3]. Weiss hypothesized that it may be due to navicular bone avascular necrosis because of the similarity of radiological findings between Keinbock disease and MWD [4]. The avascular necrosis theory has also been supported by Simons [11] and Haller et al. [6].

Maceira and Rochera hypothesized that delayed navicular ossification and an uneven distribution of compressive stress result in increased deformity and fragmentation of the cartilaginous navicular bone [7]. Weak navicular bone chondral structure caused by delayed ossification would not allow the bone to withstand compression stress, resulting in plastic deformation and an altered shape [7]. Delayed ossification could be localized only to the navicular bone or as part of a systemic condition [7]. Preconditions causing uneven navicular bone compressive forces include subtalar varus, first ray brachymetatarsia, and clubfoot [7]. These conditions increase the compressive forces through the lateral navicular half. Other theories for MWD pathogenesis include bone necrosis secondary to traumatic insult [12] and dysplasia [13]. Osteochondritis dissecans has also been suspected as a cause for MWD, but osteochondritis dissecans only affects the talar side of the navicular bone and does not cause the characteristic deformities of MWD [14].

In 1806, Scarpa introduced the concept of acetabulum pedis due to the similarity between talar head movements in the hindfoot and femoral head movements in the hip joint [15]. Acetabulum pedis is the bony socket that contains the talar head and is formed by the posterior navicular articular facet, calcaneus anterior and middle facets, the spring ligament, the superomedial calcaneonavicular ligament, and the navicular component of the bifurcate ligament [16,17]. Medial talar head displacement within the acetabulum pedis causes subtalar valgus, whereas lateral displacement causes subtalar varus [7]. Lateral navicular collapse in MWD causes a deficient lateral wall of the acetabulum pedis and subsequent lateral protrusion of the talar head and subtalar varus [7]. As MWD advances, the navicular bone fragments into medial and lateral segments, with widening gaps in between. The talar head plantarflex in the created gap results in equinus hindfoot, while the calcaneus remains inverted, creating a paradoxical pes planus varus deformity, which is characteristic of MWD [7].

Clinical presentation

The common presentation of MWD is insidious longstanding perinavicular and dorsal foot pain in patients who are in their 40s or 50s with no traumatic history [7,18,19]. Lateral ankle instability and pain around the peroneal tendons are frequent manifestations in MWD presentation [19]. Physical signs of MWD can be variable, ranging from significant midfoot pain restricting mobility to advanced foot deformity without pain or symptoms [19]. The medial arch of the foot could be preserved, flattened, or even high in combination with a hindfoot varus [7,19]. Hindfoot varus deformity may not be very explicit on inspection and may only be detected by palpating the hindfoot during heel rise [19]. Pseudo-hindfoot valgus may also be noticed because of a prominent medial navicular tuberosity [7]. A paradoxical pes planus varus (pes planus and fixed hindfoot varus) with a laterally pointed talar head is characteristic of the advanced stage of MWD [7,19]. As the navicular bone collapses and pes planus varus deformity develops, the tibia rotates externally, altering lower limb biomechanics and increasing the risk of knee arthritis [7,19].

Imaging modalities

Weight-bearing anteroposterior (AP) and lateral foot radiographs are the primary diagnostic imaging modalities for MWD [7,19-21]. Other imaging modalities include computed tomography (CT) scan, and magnetic resonance imaging (MRI) scan could be beneficial for preoperative planning and excluding other pathologies [7,20,21]. Bone scintigraphy is sensitive in detecting early changes; however, it is not specific and is rarely required in routine practice [1].

Weight-Bearing Radiographs

Weight-bearing foot and ankle X-rays are used to evaluate the extent of navicular fragmentation, deformity, and arthritic changes [7,19-22]. Weight-bearing X-rays are also beneficial for ruling out navicular bone stress fractures and differentiating MWD from talonavicular (TN) joint arthritic changes caused by rheumatoid disease or previous trauma [19-21]. MWD radiological findings can be categorized according to their anatomical location into those affecting the ankle and hindfoot and those impacting the midfoot and forefoot.

Ankle and hindfoot findings: Ankle and hindfoot findings include hindfoot varus and subtalar inversion [7,19,21]. Additionally, there could be a reduced angle between the talus longitudinal axis and the calcaneal longitudinal axis on the lateral view (the talocalcaneal divergence angle, normal range: 25°-40°), giving the impression of wide sinus tarsi [7,21]. Another finding could be the loss of the cyma line in the AP and lateral views. The cyma line is an italic S-shaped line and represents a smooth joining of the midtarsal joint lines of the TN and calcaneocuboid articulations [7,21]. Alternatively, a wide talar head appearance could be identified because of the degenerative changes and the changed coronal plane rotation [7,21]. Finally, the ankle could appear externally rotated with a retro-positioned fibula on the lateral view [7,21].

Midfoot findings: Midfoot findings can include the collapse of the posteroanterior width of the navicular bone in the lateral view. The degree of collapse varies according to disease severity. A complete collapse of the navicular bone leads to direct contact between the talar head and lateral cuneiform [7,21]. A comma or hourglass appearance of the navicular bone on the AP view could be observed and is caused by lateral collapse and sclerosis [7,21]. Alternatively, a positive cuboid sign on the AP view could be seen because of medial subluxation of the cuboid in relation to the calcaneus [7,21,23].

Forefoot findings: On the AP view, a forefoot finding could be that the metatarsal bones appear parallel to each other due to transverse arch failure [7,21]. Force bypass from the first to the second metatarsal could lead to hypertrophy of the second metatarsal [7,21]. Finally, the hallux bone may appear shorter than the second metatarsal on the AP view due to internal rotation of the medial portion of the navicular bone and retro-positioning of the first cuneiform metatarsal joint [7,21].

The radiological staging system: A radiological staging system was proposed by Maceira and Rochera in 2004 based on lateral weight-bearing radiographs and is summarized in Table 1 [7]. However, this radiological staging system for MWD does not necessarily correlate with the pain and symptom severity [7].

**Table 1:** Radiological staging of MWD. MWD: Müller–Weiss disease; CT: computed tomography; MRI: magnetic resonance imaging

Stage	Findings
I	Normal X-rays, technetium scan, CT scan, and MRI (intraosseous edema). Subtle subtalar varus could be present
II	Talar head lateral displacement causes subtalar varus
III	Navicular splitting or compression causes a reduction in the medial arch height
IV	Progressive navicular compression causes equinus hindfoot
V	Complete navicular extrusion with direct talus and cuneiform contact

Computed Tomography Scan

A CT scan allows a more detailed evaluation of the bony stock and the extent of the deformity. A CT scan can identify arthritic changes in the TN and naviculocuneiform (NC) and surrounding joints, which is necessary for preoperative planning [24-26]. On the basis of CT findings, Mayich added subtalar degeneration to MWD stage IV of the staging system introduced by Maceira [7,26]. Welck et al. introduced a novel technique to diagnose MWD using a weight-bearing CT scan [22]. Weight-bearing CT promotes a better understanding of dynamic deformities and provides more information in a load-bearing position required for reconstructive surgery planning [22].

Magnetic Resonance Imaging Scan

An MRI scan is more sensitive in picking up early changes due to its ability to detect bone marrow signal changes [1]. MRI can demonstrate early arthritic changes and adjacent joint effusion, which should be considered in preoperative planning [1]. Other differential features of MWD such as stress fractures, osteonecrosis, or infection could also be excluded with an MRI scan [1,6].

Treatment

The reported treatment ladder for MWD ranges from simple conservative measurements to multiple joint arthrodeses depending on disease stage, symptom severity, and patient activity level. Studies available for MWD treatment are either case series or cohort studies, and no controlled studies have been performed to justify the best treatment option.

Nonoperative Measurements

Nonoperative management of MWD includes optimizing weight, modification of activities, modifying footwear, and orthotics [26]. Nonoperative measurements are usually explored before considering surgical options [26]. High-impact sports such as running, football, basketball, and tennis increase midfoot strain and, hence, the shearing forces through the navicular bone [26].

Footwear modification and orthotics: The main aim of orthotics and footwear modification is to provide symptomatic relief by supporting the foot arches and reducing midfoot and TN joint motion [26]. Footwear modifications for MWD involve the incorporation of a rigid sole with a rocker bottom shoe to limit midfoot motion and enhance the transition from the first to the third rocker of the gait [19,26]. Strategies for orthoses aim to correct the pes planus varus deformity associated with MWD. Rigid insoles with medial arch support and lateral heel wedges can be adopted for patients with MWD [19,26,27]. Medial arch support intends to correct navicular bone sagging, and lateral heel wedging aims to correct subtalar supination [19].

Activities modification: Patient counseling regarding modification of their activities should be considered to mitigate MWD symptoms and control the disease progression. Activities modification in MWD management aims to avoid midfoot strain and minimize the stresses through the navicular bone [26]. High-impact sports such as running, football, basketball, and tennis cause significant midfoot strain and should be avoided [26].

Operative Treatment

Surgery is usually indicated when conservative measures to alleviate symptoms fail rather than due to progressive deformity [19]. The goals of surgical treatment are stiffening the arthritic joint(s) and restoring the medial and plantar arches [1,26]. The choice of a surgical procedure to treat MWD should be determined by the extent of the healthy navicular, talar, and cuneiform bony stock; the degree of surrounding joint arthritis; and medial arch shortening. Operative procedures specified in the literature to treat MWD include percutaneous navicular bone decompression, surgical fixation of the navicular bone, navicular bone excision, single or multiple joint fusions, and calcaneal osteotomies [26-30]. However, percutaneous decompression and internal fixation of the navicular bone are rarely indicated in MWD treatment because of advanced navicular fragmentation and surrounding joint arthritis at the time of presentation [1,30].

Joint fusions: Single (TN), dual (TN cuneiform [TNC]), and triple (subtalar, TN, and calcaneocuboid) joint fusions have been reported to treat MWD depending on the disease severity and involvement of the surrounding joints [29-32]. Both TN and TNC fusion have shown good clinical outcomes and improved quality of life for MWD stage III and IV [23,31]. However, isolated TN fusion should be considered only if osteoarthrosis is limited to the TN joint, which can be confirmed using preoperative MRI [31]. TNC has been advocated by many authors to relieve symptoms and restore the medial arch in MWD [1,30,31].

If the subtalar and/or the calcaneocuboid joint is involved, triple fusion could be a suitable option to treat MWD [32]. Triple fusion can achieve better bony consolidation in addition to medial and lateral stability [30,32]. Triple joint fusion can be performed either arthroscopically or through an open approach [32,33]. However, triple fusion does not include NC joint fusion; therefore, it can result in persistent postoperative pain if NC degenerative changes have not been properly addressed [1,7,32]. Moreover, patients may experience difficulties when walking on uneven ground after triple fusion due to the restricted hindfoot movement [32]. Triple fusion can be a suitable option for functionally low-demand patients with advanced subtalar and calcaneocuboid arthritis. Doyle et al. [8] and Zhang et al. [32] reported adding NC fusion to triple fusion in cases of advanced MWD.

Calcaneal osteotomy: Calcaneal osteotomy has been reported for hindfoot varus correction either in isolation [28] or in combination with TN fusion [29]. Li et al. reported satisfactory results after valgus calcaneal wedge osteotomy and lateral translation to correct the hindfoot varus deformity in 13 patients [28]. Additionally, Qu et al. used a combination of calcaneal osteotomy and TN fusion to treat 14 MWD patients with satisfactory results [29].

## Conclusions

MWD is a traumatic fragmentation of the navicular bone that usually occurs in adults and results in midfoot pain and deformity. The pathology of MWD remains unclear, mostly due to lateral overload of the suboptimally ossified navicular bone. The paradoxical pes planus varus deformity is characteristic of MWD. Lateral weight-bearing X-ray is the standard imaging method for diagnosis and disease staging. Treatment options range from simple conservative measures to perinavicular single or multiple joint fusions depending on symptom severity and joint involvement rather than the extent of the deformity. Multicenter randomized controlled studies are required to compare and justify the efficacy of different treatments.
